# External magnetic field-induced selective biodistribution of magnetoliposomes in mice

**DOI:** 10.1186/1556-276X-7-452

**Published:** 2012-08-10

**Authors:** Sonia García-Jimeno, Elvira Escribano, Josep Queralt, Joan Estelrich

**Affiliations:** 1 Departament de Fisicoquímica, Facultat de Farmàcia, Universitat de Barcelona, Avda. Joan XXIII, Barcelona, Catalonia, 08028, Spain; 2 Departament de Farmàcia i Tecnologia Farmacèutica, Facultat de Farmàcia, Universitat de Barcelona, Avda. Joan XXIII, Barcelona, Catalonia, 08028, Spain; 3 Departament de Fisiologia, Facultat de Farmàcia, Universitat de Barcelona, Avda. Joan XXIII, Barcelona, Catalonia, 08028, Spain; 4 Institut de Nanociència i Nanotecnologia de la Universitat de Barcelona (IN2UB), Barcelona, Catalonia, 08028, Spain

**Keywords:** magnetoliposomes, magnetic nanoparticles, inflammation, biodistribution, external magnetic field

## Abstract

This study looked at the effect of an external magnet on the biodistribution of magnetoliposomes intravenously administrated in mice (8 mg iron/kg) with and without induced acute inflammation. Our results showed that due to enhanced vascular permeability, magnetoliposomes accumulated at the site of inflammation in the absence of an external magnetic field, but the amount of iron present increased under the effect of a magnet located at the inflammation zone. This increase was dependent on the time (20 or 60 min) of exposure of the external magnetic field. It was also observed that the presence of the magnet was associated with lower amounts of iron in the liver, spleen, and plasma than was found in mice in which a magnet had not been applied. The results of this study confirm that it is possible to target drugs encapsulated in magnetic particles by means of an external magnet.

## Background

The use of materials in nanoscale provides unparallel freedom to modify fundamental properties such as solubility, diffusivity, blood circulation half-life, drug release characteristic, and immunogenicity. In the last two decades, nanoparticles have attracted widespread attention due to the rapidly increasing number and variety of their applications in the biomedical sciences, including imaging and therapy [1,2]. Indeed, they possess unique nanoscale size-dependent physical and chemical properties that can be controlled in a manner that is not possible with bulk materials [3]. There are several nanoparticle platforms: liposomes, polymeric conjugates, polymeric nanoparticles, micelles, nanoshells, dendrimers, engineered viral nanoparticles, albumin-based nanoparticles, polysaccharide-based nanoparticles, ceramic nanoparticles, and magnetic nanoparticles.

Among the aforementioned particles, iron oxide-based magnetic nanoparticles (MNs) have been developed and represent promising new systems in biomedical sciences, especially in therapy and imaging [4]. Medical applications of MNs require that such nanoparticles possess superparamagnetic properties at room temperature. This property is displayed in small subdomain particles (of the order of tens of nanometers or less; 10 to 15 nm for magnetite). Superparamagnetic particles have fast relaxation times and, consequently, show neither remanence nor coercivity. Thus, these nanoparticles are nonmagnetic in the absence of an external magnetic field, but they do develop a mean magnetic moment in an external magnetic field [5].

Therapeutic application of MNs is when they are used for the site-specific delivery of therapeutic payloads. The goal of any drug delivery system is to ensure that the drugs are administered into the body correctly and reach the target area. Targeting a drug delivery system can be divided into two general categories: passive and active. Although both approaches are used, the technical demands of the two are considerably different. Passive targeting exploits the natural distribution pattern, i.e., the tendency of unmodified particles to accumulate in certain tissues, which occurs because nanoparticles are quickly removed from circulation by the cells of the mononuclear phagocyte system (MPS), in particular, the resident macrophages of the liver (Kupfer cells), spleen, lung, and bone marrow [6]. Hence, this strategy serves to target diseases that affect the MPS. Active targeting involves modifying the basic structure of nanoparticles by attaching a homing device to the carrier. Examples of particle modifications include the attachment of ligands such as antibodies - which allow it to complex with the cell that contains the receptor for the interaction - and the incorporation of molecules that make the particles susceptible to pH or temperature changes [7].

MNs share the main advantages of both types of targeting; as with passive targeting, modification of the nanoparticle surface is not necessary, and like active targeting, they can be directed to the area of interest. This is due to the fact that MNs respond strongly to time-modulated magnetic fields, and in turn, magnetic fields can penetrate human tissues without impediment. Thus, MNs can be used as a tool for targeting drugs, such as anticancer or radionuclide atoms, which could be co-encapsulated within the magnetic nanoparticles and then carried within the nanoparticle structure under the influence of an external magnetic field to a targeted area in the human body. As with MNs, magnetoliposomes (MLs) formed by encapsulating iron oxide nanoparticles within liposomes have the advantage of being nontoxic and biocompatible and displaying magnetophoretic mobility [8,9]. Magnetoliposomes play an important role in the wide variety of MNs, which are routinely used as magnetic resonance imaging contrast agents [10,11] and for controlled drug release [12].

In this paper, we assess the effect of a magnetic field, generated outside the body, on the biodistribution of magnetoliposomes after intravenous administration in mice to which an inflammatory focus on their back has been induced. Cancer and inflammation are related by epidemiology, histopathology, and inflammatory profiles [13]. For this reason, the development of an experimental model to study *in vivo* whether the drug correctly reaches and concentrates at the target site is of great interest. The distribution of MNs within different organs and timescales (20 and 60 min) was examined by determining the amounts of the iron accumulated in the tissues.

## Methods

### Materials

Soybean phosphatidylcholine (PC), a zwitterionic phospholipid (Lipoid S-100), was donated by Lipoid (Ludwigshafen, Germany). Nanoparticles of magnetite stabilized with anionic coating (EMG 707) were purchased from FerroTec (Bedford, NH, USA). The particles had a nominal diameter of 10 nm, which was determined by transmission electron microscopy (TEM); a stock solution has a coefficient of viscosity of less than 5 mPa·s at 27°C and a magnetite volume content of 1.8%. Neodymium-iron-boron (Nd_2_Fe_12_B) magnet discs (N35D2510, 25 × 10 mm, of 600 mT by side) were obtained from Halde GAC (Barcelona, Spain). Reagents were of analytical grade.

### Preparation and characterization of magnetoliposomes

Magnetoliposomes were obtained using a modified version of the phase-reverse method followed by extrusion at room temperature into a Liposofast device (Avestin, Inc., Ottawa, Canada) through two polycarbonate membrane filters of 0.2-μm pore size, a minimum of nine times both ways [14]. Ferrofluid was diluted with 0.16 M NaCl until an iron concentration of 1.12 mg mL^−1^ was achieved, and with this aqueous suspension, the phospholipid layers were hydrated. Ferrofluid particles no longer entrapped were removed by size exclusion chromatography on Sepharose 4B (GE Healthcare, Chalfont Saint Giles, UK). A total of 250 μL of magnetoliposomes was applied to a 1 × 30-cm column saturated with lipids before sample elution with 0.16 M NaCl. Magnetoliposomes were then separated from empty liposomes by means of a MACS separation column (Miltenyi Biotech, Bergisch Gladbach, Germany). The morphology of magnetoliposomes was observed by TEM using a JEOL 1010 microscope (JEOL Ltd., Akishima, Tokyo, Japan) operating at 80,000 V. A drop of the aqueous dispersion of magnetoliposomes was placed onto a 400-mesh copper grid coated with a carbon film with a Formvar membrane and then allowed to air dry before being inserted into the microscope. Images were recorded with a Megaview III camera (Arecont Vision, Glendale, CA, USA), and the acquisition was accomplished using Soft-Imaging software (SIS, Münster, Germany). The iron content of the magnetic particles was determined on the basis of the ferrous ion using *o*-phenanthroline [15]. The phospholipid content was determined by the Stewart-Marshall method [16]. The hydrodynamic diameter of magnetoliposomes was determined by dynamic light scattering (DLS) at 90° with the Zetasizer Nano (Malvern Instruments Ltd., Malvern, Worcestershire, UK) at a temperature of 25°C. The particle size distribution was designated by the polydispersity index (PI), which ranged from 0.0 for an entirely monodisperse sample to 1.0 for a polydisperse sample. The ζ-potential measurements of magnetoliposomes were performed at 25°C using the Zetasizer Nano ZS (Malvern Instruments Ltd., Malvern, Worcestershire, UK).

### Animal and biological methods

Experiments were performed on 8-week-old female CD-1 mice with a body weight of 28 to 32 g (Harlam Ibérica, Sant Feliu de Codines, Barcelona, Spain). Animals were provided with food and tap water *ad libitum* and allowed a 1-week acclimation period after arrival to the animal facilities of the Faculty of Pharmacy. The animals were then allowed an additional week to adjust to restraining conditions. The study was conducted under a protocol approved by the Animal Experimentation Ethics Committee of the University of Barcelona, Barcelona, Spain.

Mice were randomly divided into four groups (between seven and eight animals per group): (a) healthy control saline (C): animals with back air pouch receiving saline i.v. (0.1 mL/10 g body weight); (b) inflammation control group (I): animals with carrageenan-induced inflammation in the air pouch receiving saline i.v.; (c) inflammation magnetoliposomes (IN): animals with carrageenan-induced inflammation receiving magnetoliposomes i.v.; and (d) inflammation magnetoliposomes with magnet (INM): as in the previous group (IN), but under the effect of a magnet located just above the inflammatory pouch for 20 or 60 min. All injections and painful procedures were performed under isoflurane (Laboratorios Esteve, Barcelona, Spain) anesthesia (4% induction and 3% maintenance).

The method used to induce acute inflammation was similar to that described by Romano et al. [17]. Briefly, sterile air pouches were produced by injecting 5 mL of air subcutaneously (s.c.) through a disposable disc filter (EPS® Inc., Ivyland, PA, USA; 2 μm) into the back of the mouse (day 0); the pouches were injected again with 3 mL of air on day 3. On day 4, the pouches from the mice in group C received 1-mL saline (B Braum Medical, Barcelona, Spain), and the pouches from the mice in groups I, IN, and INM received 1-mL carrageenan lambda 1.2% s.c. (Sigma-Aldrich, St. Louis, MO, USA).

On day 5, 24 h after the phlogogen injection, the animals received 0.1 mL/30 g saline (group C) or magnetoliposomes (8 mg of iron/kg body weight) in saline (groups I, IN, and INM) i.v. through a jugular vein and were placed in restrained Plexiglas cages with air holes. Some mice did not have disc magnets on their backs (groups C, I, and IN) while some did for 20 or 60 min. Before i.v. administration, a small incision was made in the neck to one side of the midline. A 30-G needle was used for the injection (Becton Dickinson D Ultra-Fine U-100 insulin Syringe, USA) and was inserted into the vein through the pectoral muscle at an approximate angle of 10°, pointing toward the head. The muscle acts as a seal when the needle is withdrawn. To close the skin incision, one or two sterile silk suture 4/0 points were applied (Laboratorios Aragó, Barcelona, Spain).

**Figure 1:**
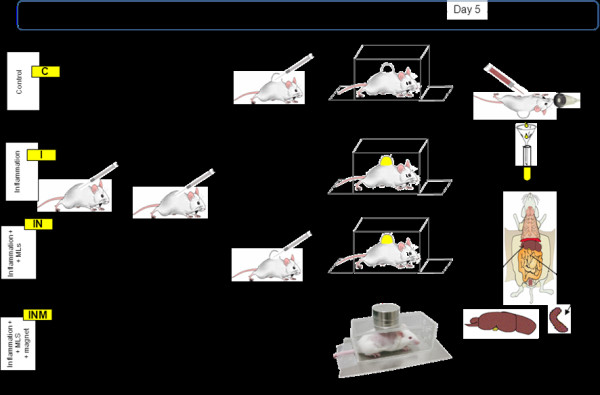
Experimental procedure performed to establish the effect of external magnet on biodistribution of MLs.

After 20 or 60 min of i.v. dosing, mice were anesthetized, thoracotomy was performed, and the blood was harvested by cardiac puncture using a 27-G Becton Dickinson tuberculin syringe with needle. Plasma was obtained by centrifugation (10,000 × *g* for 5 min) (Heraeus Biofuge Pico, Hanau, Germany). When the heart stopped beating, 2.5 mL of ultrapure water (Purelab® ultra analytics system, ELGA, Veolia Water, Barcelona, Spain) was injected into the pouch. The pouch was gently massaged, and the mouse was placed in the supine position on the top of a plastic funnel. An incision was made in the pouch, and the exudates were collected in 5-mL polyethylene tubes and weighed. The liver and spleen were harvested and dried by placing the samples in a laboratory heater at 60°C for 24 h. Figure 1 shows a schematic of the experimental procedure carried out.

### Analytical determinations

To control inflammation, the volume (in milliliters) and protein content (in milligrams per milliliter) of the exudate were determined. The volume was determined by weighing the exudates, and the protein content was measured using the bicinchoninic acid assay [18]. The iron content in the plasma, exudates (pouch), and organs was analyzed by inductively coupled plasma optical emission spectrometry (Perkin-Elmer Optima 3200RL, Massachusetts, USA) after acid digestion of the organic material in closed systems. Microwave digestion (210°C, 1 h) was performed on organs, and digestion for plasma and exudates was carried out in Teflon vessels (90°C, 24 h).

The results are expressed as micrograms of iron per gram of dried tissue. The statistical significance of iron concentrations was analyzed using ANOVA followed by the post hoc Fisher’s LSD test. Statistical significance was accepted at the 5% level (*P* ≤ 0.05).

The presence of magnetoliposomes in the exudate was observed by TEM. To obtain the magnetoliposomes from the exudate, the exudate was eluted through the MACS separation column, and a drop of the aqueous dispersion after air drying was observed into the microscope as previously explained.

## Results

**Figure 2:**
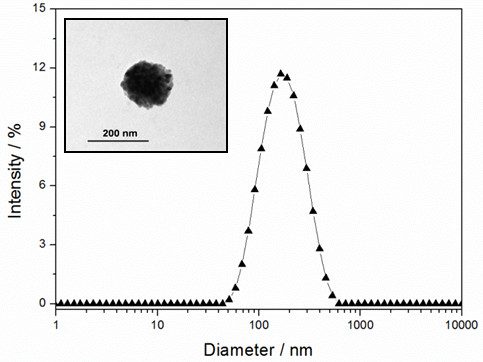
**Size distribution expressed as*z*-diameter of magnetoliposomes after purification by size exclusion chromatography.** Inset: TEM micrograph of non-stained magnetoliposomes.

The obtained magnetoliposomes contain 0.135 ± 0.005 g Fe^3+^ per mmol of phospholipid. The size of the magnetoliposomes determined by DLS ranged from 150 to 190 nm (Figure 2) with a single peak centered at 183 nm. This is consistent with our earlier results [12]. As can be observed, the magnetoliposomes are arranged in a monomodal distribution (PI < 0.2). The MLs captured within the phospholipid bilayer appear as clusters of ferrofluid particles (inset of Figure 2). At pH 7.4, a ζ-potential value of −23.5 ± 2.4 mV was obtained. This negative charge is conferred by the external phospholipid monolayer. Although PC is electrically neutral, the distribution of charges in its polar head region is asymmetrical. The negative ζ-potential of the liposomes is caused by the orientation of PC molecules in the liposome membrane.

The tissue distribution of iron oxide entrapped in magnetoliposomes and injected intravenously into mice from the four groups was studied 20 and/or 60 min after the injection. The presence of magnetoliposomes in the exudate is due to the hyperpermeability of the microvasculature in the inflammatory process. Therefore, in order to evaluate the biodistribution of magnetoliposomes, only those mice that demonstrated a protein concentration in exudates between 3.5 and 8.5 mg mL^−1^ as well as a similar volume of exudates for the animals induced with inflammation (2.6 ± 0.2 mL) were considered Values from 0.2 to 1.0 mg mL^−1^ are characteristic of an absence of inflammation.

Tables 1 and 2 summarize the results obtained for volume and protein content in the pouch, as well as for the iron content in the exudates, plasma, liver, and spleen 20 and 60 min after the injection of magnetoliposomes. The amount of iron determined in biological liquids and organs was higher after 60 min than after 20 min. The absence of a magnet resulted in a preferential accumulation of magnetoliposomes in the spleen and liver, consistent with the uptake of such particles by the mononuclear phagocyte system. The presence of the magnet (INM) resulted in a decrease in the iron present in the liver (*P* < 0.05) in comparison with group IN (a reduction of 23% after 20 min of the injection and of 25% after 60 min). In the plasma, the external magnet provoked a significant reduction of 30% and 31% for 20 and 60 min, respectively (*P* < 0.05). In the spleen, the reduction was less pronounced (13% and 16% for 20 and 60 min, respectively). Such reductions can be explained by the increase in iron content in the exudates. Table 1 shows how the levels of iron in the inflammatory focus increased 1.7 times (after 20 min) and three times (after 60 min) after the injection.

**Table 1:** Volume and protein content of the exudates and iron concentration in exudates, plasma, liver, and spleen

**Groups**	**Pouch**		**Iron concentration (μg g**^**−1**^**)**			
	**Volume (mL)**	**Protein (mg mL**^**−1**^**)**	**Exudates**	**Plasma**	**Liver**	**Spleen**
C	1.96 ± 0.24	0.61 ± 0.37	0.04 ± 0.04	4.04 ± 1.02	621 ± 310	665 ± 180
I	2.38 ± 0.33^a^	5.69 ± 2.01^a^	0.26 ± 0.13^a^	4.01 ± 1.27	712 ± 224	836 ± 278
IN	2.74 ± 0.24^a^	5.23 ± 1.43^a^	0.33 ± 0.11^a^	4.12 ± 1.07	873 ± 99^a^	1,446 ± 390^a,b^
INM	2.57 ± 0.05^a^	4.63 ± 0.21^a^	0.56 ± 0.12^a,b,c^	2.89 ± 0.42	674 ± 64^c^	1,264 ± 35^a,b^

The values are expressed as mean ± SD and were obtained 20 min after the injection of magnetoliposomes. ^a,b,c^ Significant differences (*P* < 0.05) with groups C, I, and IN, respectively (*n* ≥ 5).

**Table 2:** Volume and protein content of the exudates and iron concentration in exudates, plasma, liver, and spleen

**Groups**	**Pouch**		**Iron concentration (μg g**^**−1**^**)**			
	**Volume (mL)**	**Protein (mg mL**^**−1**^**)**	**Exudates**	**Plasma**	**Liver**	**Spleen**
C	2.14 ± 0.02	0.63 ± 0.20	0.19 ± 0.12	4.45 ± 0.33	547 ± 91	914 ± 403
I	2.80 ± 0.23^a^	5.90 ± 2.53^a^	0.50 ± 0.10	4.12 ± 0.68	605 ± 114	1,039 ± 227
IN	2.85 ± 0.13^a^	5.20 ± 1.60^a^	0.64 ± 0.27	4.25 ± 1.43	1,174 ± 134^a,b,c^	1,611 ± 378^a,b^
INM	2.77 ± 0.18^a^	4.95 ± 1.01^a^	1.92 ± 1.44^a,b,c^	2.94 ± 0.38^a,b,c^	880 ± 97^a,b,c^	1,354 ± 260^a,b^

Values are expressed as mean ± SD and were obtained 60 min after the injection of magnetoliposomes. ^a,b,c^ Significant differences (*P* < 0.05) with groups C, I, and IN, respectively (*n* ≥ 5).

As we have considered that the biodistribution of the MNs was in equilibrium with the distribution of iron, an increase in iron concentration in exudates in the IM and IMN groups was attributed to the presence of MNs in the target. To confirm this hypothesis, we determined whether MNs were present in exudates by visualizing them using TEM.

**Figure 3:**
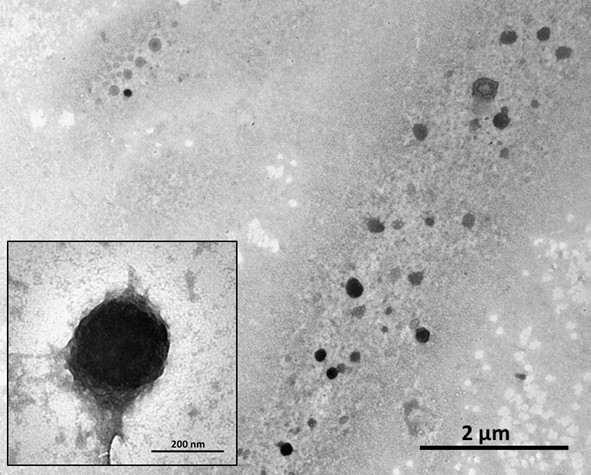
**TEM micrograph of non-stained magnetoliposomes found in exudates of a mouse (IMN).** The magnetoliposomes were found 20 min after intravenously injecting the particles. Inset: an enlarged image of a magnetoliposome.

To determine whether magnetoliposomes were present in exudates, we separated them from the liquid using a magnetic separator. They can also be separated by centrifugation due to the high density of such liposomes, caused by iron loading. However, the magnetic separator allows us to concentrate on them. After this, they were visualized using TEM. Figure 3 shows the presence of magnetoliposomes in the exudates. The majority of the magnetoliposomes maintained their original form and size.

## Discussion

The aim of this study was to determine whether magnetoliposomes could be directed *in vivo* to target sites under the effect of an external magnet, consequently avoiding modification of the liposomes. There are reports in the literature that this external stimulus actively locates and accumulates the drug at the targeted region [19]. It is known that liposomes (and therefore magnetoliposomes) can be directed through the functionalization of the external layer. For instance, liposomes with the RGD peptide exposed on their surface are able to bind vascular endothelial cells at inflammation sites [20]. The inflammatory reaction is one of the body’s defense mechanisms. When damage occurs, blood flow is firstly increased at the point of injury, and blood vessels widen to allow oxygen, clotting agents, and white blood cells to penetrate the damaged tissues. Since the size of white blood cells is ranging from 7 to 20 μm, any nanoparticles can easily pass through the vessels and locate in the inflammation zone. To achieve an inflammation zone, we used mice with induced air pouches. Among many animal models of inflammation (sponge implant, paw edema, peritoneal, pleurisy, and air pouch), the air pouch model has the advantage of not involving internal organs, which can be damaged or perforated during sampling [21]. On the other hand, we have used a strong magnet (600 mT of surface strength of magnetic field) to assure that the magnetic field lines are penetrating the body deeply, that is, the technique is not only applicable for tissues located at the body’s surface. However, the strength of the magnetic field falls off very quickly as the surface of the magnet is moved away from the target area. Besides the material of the magnet, the mass of the magnet has an important effect on its overall strength. The larger the mass of the magnet, the stronger and more effective (more uniform magnetic field) it will be. For this reason, we have used two magnets above one another (see Figure 1).

## Conclusions

By using this approach (model of inflammation and strong magnet) and excluding animals with levels of iron and volumes of exudates out of the range of average values observed during inflammation, we secured a fairly homogeneous population in which to study the effect of an external magnetic field on the distribution of magnetoliposomes after injecting them intravenously. We observed the accumulation of magnetoliposomes at the site of inflammation (exudates) and, at the same time, the removal from the blood compartment, and the decrease in the liver and spleen. As shown in Tables 1 and 2, the increase in iron levels in exudates depends on the time of exposure of the external magnetic field; when comparing IN and INM groups, an increase of 70% and 200% was observed for 20 and 60 min, respectively. After this point, the magnetic field efficiently retains MNs. This method can also be used to enhance the accumulation of a certain drug (i.e., corticosteroid, hyaluronic acid) after an intra-articular injection, thus avoiding the need of repeated injections due to rapid clearance from the joint [22]. The fact that magnetoliposomes remain intact in the exudates 20 min after the injection is not surprising since with a diameter close to 12 nm, the magnetite particles are too large to passively cross the phospholipid bilayer. Only after the action of destabilizing agents, such as opsonins and cells of the mononuclear phagocyte system, the iron content may leave the interior of the magnetoliposomes. In conclusion, this study has demonstrated the efficacy of the magnet method. We have shown that when loaded with a suitable therapeutic agent, an anti-inflammatory in this case, magnetoliposomes, under the effect of a magnetic field can be used to treat the inflammatory process or other pathologies, and in doing so, it can reduce the drug dose administered and increase the efficacy of the treatment. Moreover, the observed reduction in the levels in the blood could prevent side effects.

## Competing interests

The authors declare that they have no competing interests.

## Authors’ contributions

SD-J carried out the nanoparticle synthesis, participated in the biodistribution study, and performed the analysis of the samples. EE participated in the design of the study and performed the biological and analytical determinations. JQ participated in the design of the study, carried out the animal and biological studies, and contributed to the interpretation of data. JE participated in the design of the study and drafted the manuscript. All authors read and approved the final manuscript.
